# ^17^O Quadrupole Coupling Constants in Water

**DOI:** 10.1002/mrc.70129

**Published:** 2026-06-30

**Authors:** Nabeeha Haque, Alexander G. de Dios, Angel C. de Dios

**Affiliations:** ^1^ Department of Chemistry Georgetown University Washington DC USA

**Keywords:** ^17^O NMR, ab initio calculations, electric field gradient, hydrogen‐bonding, ice, quadrupole coupling constants, water, water clusters

## Abstract

An additive framework is developed to describe ^17^O quadrupole coupling constants (QCC's) in hydrogen‐bonded water systems across isolated molecules, finite clusters, and ice‐like environments. Density functional theory calculations using optimized geometries show that ^17^O QCC variations are primarily governed by hydrogen‐bond interactions. A dimer model identifies hydrogen‐bond distance as the dominant geometric factor, while angular and intramolecular distortions are minor. In ice and water clusters, first‐nearest‐neighbor interactions provide the largest contribution and are well captured by an additive donor–acceptor scheme, with improved accuracy upon inclusion of hydrogen bond distance dependence. Second nearest neighbor effects introduce smaller but systematic negative corrections arising from hydrogen‐bond network asymmetry, restoring near‐quantitative agreement with full calculations. The resulting model provides a computationally efficient and physically interpretable approach for predicting electric field gradients in aqueous environments and linking molecular simulations with ^17^O NMR observables.

## Graphical abstract

In water clusters, first‐nearest‐neighbor interactions (1A and 1D) provide the largest contribution and are well captured by an additive donor–acceptor scheme, with improved accuracy upon inclusion of hydrogen bond distance dependence. Second nearest neighbor (D and A*) effects introduce smaller but systematic negative corrections, restoring near‐quantitative agreement with full calculations for the ^17^O quadrupole coupling constant at the site marked with a star.

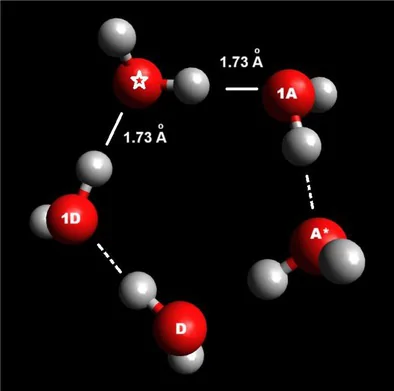

## 1 Introduction

Hydrogen bonding significantly influences the ^17^O NMR chemical shift in water [1]. Equally useful in characterizing intermolecular interactions of water in the solid state is the ^17^O quadrupole coupling constant (QCC) [2]. ^17^O NMR is indeed a powerful experimental probe that can directly quantify and measure electronic properties, and in the solid state, restricted molecular motion resulting in the absence of rotational diffusion allows for the measurement of tensor properties for this NMR parameter [3]. In addition, with partial alignment, it now has been demonstrated that even in solution phase, residual ^17^O QCC's can likewise be measured [4]. Indirect measurement of ^17^O QCC's has also long been performed with relaxation measurements [5]. Water plays a fundamental role in a wide range of chemical, biological, and environmental processes [6], therefore, understanding its interactions is of prime importance. Of course, the unique physical and chemical properties of water largely come from hydrogen bonding, which in turn influences the structural and electronic environment of water molecules in condensed phases. Understanding how hydrogen bonding alters the electronic structure of oxygen atoms is therefore essential for interpreting the behavior of water in different environments, including molecular clusters, bulk liquid, and interfacial systems.

Molecular dynamics (MD) simulations provide extensive structural sampling of aqueous environments through configurational snapshots of the molecules and have shown correlations with hydrogen bonding dynamics [7]. Hydrogen bonding dramatically changes the charge distribution felt at the site of the ^17^O nucleus, thereby affecting its electric field gradient (EFG), which then determines the QCC, the interaction between the EFG and the nuclear quadrupole moment. Previous studies have demonstrated that the QCC is a sensitive probe of how hydrogen bonding perturbs the electron distribution in a range of environments [8, 9, 10, 11, 12]. Verhoeven and co‐workers have observed an experimental value, 10.17 ± 0.07 MHz, for the QCC of an isolated water molecule, which provides a reference for computational studies of hydrogen bonding effects on the ^17^O QCC in water [13]. For water molecules in the liquid and gaseous states, experimental values have been previously compared to calculations using density functional theory (DFT). The QCC's computationally obtained for the gas phase is 10.75 MHz and for the liquid, 7.77 MHz [14]. Quadrupole resonance measurements have been performed on ^17^O NMR in ice, with the experimental value being 6.525 ± 0.015 MHz [15]. DFT calculations using a moderately sized basis set favorably agree with experimental values using a cluster consisting of 42 water molecules, producing a value of 6.73 MHz for water in the solid state [15]. These two benchmark systems demonstrate the ability of the DFT method to extract quantitatively the effects of hydrogen bonding on the ^17^O quadrupole coupling constant in water.

As the size of a system increases, it is obvious that the ^17^O QCC values of water molecules decrease. Finite water clusters optimized to minimum‐energy structures can therefore be utilized as models for studying hydrogen bonding and for generating configurations in MD simulations for subsequent NMR parameter calculations [16, 17, 18]. Within a water cluster, the nearest neighbors have had marked effects on the QCC values for the central water molecule depending on the phase. For example, a cluster consisting of five water molecules forming a tetrahedral structure around the central water molecule produces predictable downshifts for liquid water, but the decrease is still much less compared to experimental values for ice structures [19]. However, despite improving the agreement between calculated and experimental values with increasing cluster size, this comes with a price as the computational time required for these calculations becomes consequentially more demanding. This work aims to provide an analytical way of calculating QCC's for ^17^O in water without the use of molecular clusters. Such an approach is similar to what has been recently demonstrated for ^17^O NMR chemical shifts [1].

The dependence of water's ^17^O QCC's on hydrogen bonding can be analyzed by changes in the internal geometry of the water molecule. In addition, hydrogen bond distances and angles need to be examined. Furthermore, whether a water molecule is acting as an acceptor or donor may also matter. This work aims to arrive at a quantitative and transferable relationship between local hydrogen‐bond geometry and the QCC of ^17^O in water. This work investigates whether the geometric effects of multiple hydrogen bonds can be treated as additive contributions from individual neighbors, thereby providing a framework for predicting the EFG for water molecules in any environment. If hydrogen bond contributions to the EFG are approximately additive, QCC's for water could be predicted for every configuration in an MD trajectory. This would allow large‐scale simulations to be directly compared with quadrupolar NMR experiments.

## 2 Results and Discussion

### 2.1 Basis Set Dependence

A saturated basis set is necessary to reproduce accurately an electronic property such as the EFG at the ^17^O nucleus. An analysis of the basis set dependence of calculated QCC's of ^17^O in an isolated water molecule is performed with augmented (with diffuse functions) Dunning's correlation consistent basis sets; aug‐cc‐pVDZ [20], aug‐cc‐pVTZ [21], aug‐cc‐pVQZ [22], and aug‐cc‐pV5Z [23]. Smaller basis sets like the augmented correlation consistent with polarization double zeta basis (aug‐cc‐pVDZ) use a limited number of functions to approximate atomic orbitals and therefore only consider one virtual shell. Larger basis sets like aug‐cc‐pVTZ include additional functions for greater flexibility in representing electron density, which improves the accuracy of the results, and increasing the basis set size to QZ or 5Z would improve the accuracy even more. However, larger basis sets are more computationally expensive and thus a saturation of the basis set is desired to approximate accurate QCC values with manageable computational time, similar to what has been done for the ^17^O NMR chemical shift in water [24]. Comparison of isolated water molecules and ice structure QCC's shows that although they both continue to increase with increasing basis set size, the difference between the two systems becomes significantly more stable, past the TZ level. The difference between the QCC in isolated water and ice becomes monotonic (see Table 1). Furthermore, TZ is the largest basis set with a reasonable computational time (CPU) of 6 h for (H_2_O)_17_ compared to QZ which requires a CPU time of 2 days and 15 h.

**TABLE 1:** Basis set dependence of ^17^O QCC for isolated water and (H_2_O)_17_ ice cluster^a^.

Basis set	H_2_O, MHz	(H_2_O)_17_, MHz	ΔQCC (Water–Ice), MHz
aug‐cc‐pVDZ	10.20	6.90	3.30
aug‐cc‐pVTZ	10.34	7.16	3.18
aug‐cc‐pVQZ	10.58	7.37	3.21
aug‐cc‐pV5Z	10.67	7.40	3.27

^a^ CPU times for (H_2_O)_17_: DZ (44 min), TZ (6 h), QZ (2 d, 15 h), and 5Z (17 d, 20 h).

The calculated QCC's with the aug‐cc‐pVTZ basis are 10.34 MHz for isolated water and 7.16 MHz for ice, compared to the experimental values of 10.17 MHz [13] and 6.66 MHz [15], respectively. With increasing basis set size, the calculated values actually become farther from the experiment for both isolated water and ice, indicating that a higher level of theory is necessary. However, increasing the size of the basis does make the difference between ice and isolated water closer to experiment. Unfortunately, a higher level of theory is currently prohibitive with present computational resources. All of the computations reported in this paper are therefore done with the hybrid density functional B3LYP [25, 26]. Nevertheless, the results using the aug‐cc‐pVTZ (TZ basis) basis set correspond to deviations of 1.6% for isolated water and 7.5% for ice, with the calculated water–ice difference (3.18 MHz) within 10% of the experimental value (3.52 MHz), demonstrating that the chosen basis set and cluster size can reproduce experimental trends with acceptable accuracy. Lastly, it should also be noted that none of these calculations include vibrational averaging.

### 2.2 Cluster Size Dependence

The cluster size of (H_2_O)_17_ was chosen since, although the 5‐water cluster contains the first hydration sphere and the directly H‐bonded molecules, the 17‐water cluster model more correctly exhibits hydrogen bonding in ice with three molecules (second‐nearest neighbors) hydrogen bonded to each of the outer molecules in the first sphere [24]. Additionally, while the 42‐water cluster forms a third hydration sphere that fills in the cavities, with the TZ basis set, it is already much more computationally expensive than the 17‐water cluster, which already shows a convergence of QCC values at a shorter CPU time (Table 2).

**TABLE 2:** Dependence of ^17^O QCC and computational time on cluster size with TZ basis.

Molecule	QCC, MHz	CPU time, minutes
H_2_O	10.34	0.18
(H_2_O)_5_	7.71	9
(H_2_O)_17_	7.16	971
(H_2_O)_42_	7.04	24,180

A further analysis of nearest‐neighbor contributions supports the use of the (H_2_O)_17_ cluster size for the computation of QCC values in H‐bonding environments. It is demonstrated that the nearest neighbors contribute significantly while the second nearest neighbors already contribute much less (Table 3). For both acceptor and donor molecules, changes in the QCC are now an order of magnitude less, confirming that interactions beyond the second hydration sphere are only expected to be much smaller and that the 17‐water cluster already provides a converged model of the electronic environment in ice. It is likewise noteworthy that the TZ and QZ basis sets lead to almost equivalent results for both first and second neighbor effects.

**TABLE 3:** Basis set dependence of first and second nearest neighbor contributions to ^17^O QCC.

Interaction type	TZ, MHz	QZ, MHz
**First nearest neighbors** ^**a**^		
A	−1.13	−1.14
D	−0.30	−0.31
**Second nearest neighbors** ^**b**^		
A*	−0.13	−0.14
D*	0.13	0.13
A	0.10	0.09
D	−0.10	−0.11

^a^ A and D denote acceptors and donors to the central water molecule, respectively.

^b^ A* and D* denote acceptors and donors to a first‐neighbor acceptor molecule, respectively. A and D denote acceptors and donors to a first‐neighbor donor molecule, respectively.

### 2.3 Dimer Model Predictions of QCC Dependence on Hydrogen Bonding Geometry

To establish a predictive relationship between hydrogen bonding and the ^17^O QCC, it is necessary to isolate how the geometry of individual hydrogen bonds influences the EFG. As hydrogen bonding can alter the internal geometry of a water molecule, geometry optimization is performed at the MP2 level of theory [27]. Using the calculated QCC of 10.34 MHz for an isolated water molecule with geometry optimized at MP2/aug‐cc‐pVTZ as reference, the individual effects of both a hydrogen bond donor and acceptor molecule are determined using a dimer model (Figure 1) to provide a controlled system in which a single hydrogen bond can be examined independently of competing interactions present in larger clusters.

**FIGURE 1:**
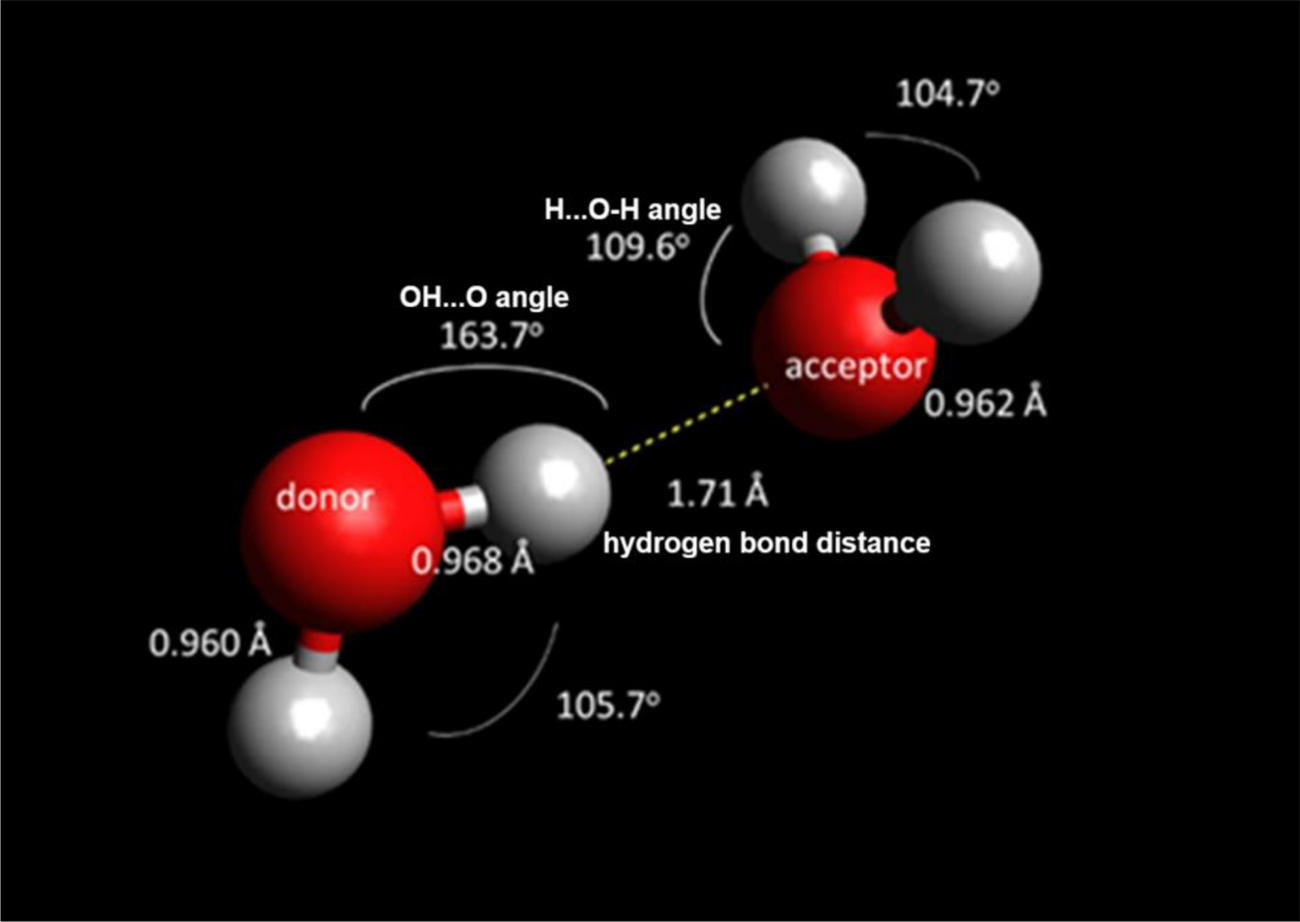
The model water dimer used in the ^17^O QCC computations.

When a water molecule acts as a hydrogen bond donor, the QCC exhibits an approximately 1/*r* ^4^ dependence with respect to the HO⋯H hydrogen bond distance (see Figure 2a). This hydrogen bond distance is the separation in Angstrom units (Å) between the O atom of an acceptor water molecule and the H atom of a donor water molecule. At the optimized geometry, this distance is 1.71 Å (see Figure 1). Such behavior is consistent with the expected response of the electric field gradient of the ^17^O nucleus of one water molecule to a dipolar interaction, where the gradient decays more rapidly than the corresponding electric field provided by the second water molecule.

**FIGURE 2:**
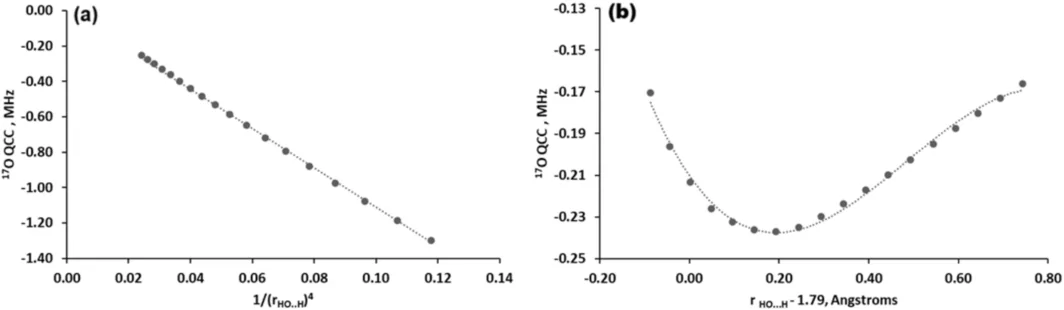
^17^O QCC dependence on hydrogen bond distance in a dimer model. (a) Donor case showing ~1/*r* ^4^ behavior. (b) Acceptor case showing weak distance dependence.

In contrast, when the water molecule acts as a hydrogen bond acceptor, the QCC shows a weak and nonmonotonic dependence on hydrogen bond distance (Figure 2b), characterized by an initial decrease with increasing distance, followed by an increase at larger separations. At short hydrogen bond distances, the orbital interaction dominates and strongly perturbs the anisotropy of the oxygen electron density, producing significant changes in the EFG. This is not surprising as the nonbonding orbitals on oxygen dramatically change when the O atom begins to accept a hydrogen bond. This dependence is opposite in sign compared to a simple effect from a dipole, that is, a decreasing EFG with increasing hydrogen bond distance. As the distance increases, this interaction rapidly diminishes due to the localized nature of orbital overlap, and the residual long‐range electrostatic contribution becomes dominant, giving rise to the expected increase in the QCC with increasing separation. This resulting nonmonotonic behavior indicates that hydrogen bond acceptor interactions at oxygen are driven by a combination of orbital overlap effects and classical electrostatic polarization, rather than a single distance‐dependent interaction. With these dimer calculations, changes in QCC can be easily estimated from just the hydrogen bond distance. The ΔQCC (change in quadrupole constant with respect to an isolated molecule) dependence on the hydrogen bond distance *r* (OH …. O (in Å)) is given by the following equations (where the labels acceptor and donor correspond to the role of the first neighbor):

(1) $$\text{Acceptor}:\text{ΔQCC}\;\left(\mathrm{MHz}\right)=-11.11\;\left(\frac{1}{r^{4}}\right)$$

(2) $$\begin{array}{c} \text{Donor}:\text{ΔQCC}\;\left(\mathrm{MHz}\right)=-0.69\;{\left(r-1.79\right)}^{3}+{\left(r-1.79\right)}^{2}\\ -0.31\left(r-1.79\right)-0.21 \end{array}$$

In an H⋯OH hydrogen bond, the dependence of the ^17^O quadrupole coupling constant (QCC) on the angle of approach of the hydrogen atom toward the oxygen lone pairs is likewise examined. For the hydrogen bond acceptor molecule, a systematic increase in the QCC is observed with increasing H⋯O‐H interaction angle, indicating a measurable angular dependence associated with the orientation of the hydrogen bond relative to the oxygen lone pair density (Figure 3a). In contrast, the donor molecule showed only a weak or negligible dependence of the QCC on this angle (Figure 3b). For both the acceptor (Figure 3c) and donor (Figure 3d) molecules, the OH⋯O hydrogen bond angle, corresponding to rotation of the hydrogen bond axis, is also studied. In both cases, the QCC exhibits a slight decrease with increasing hydrogen bond angle, consistent with reduced hydrogen bond alignment. However, in all cases, the magnitude of this variation is small within the large range of angles considered.

**FIGURE 3:**
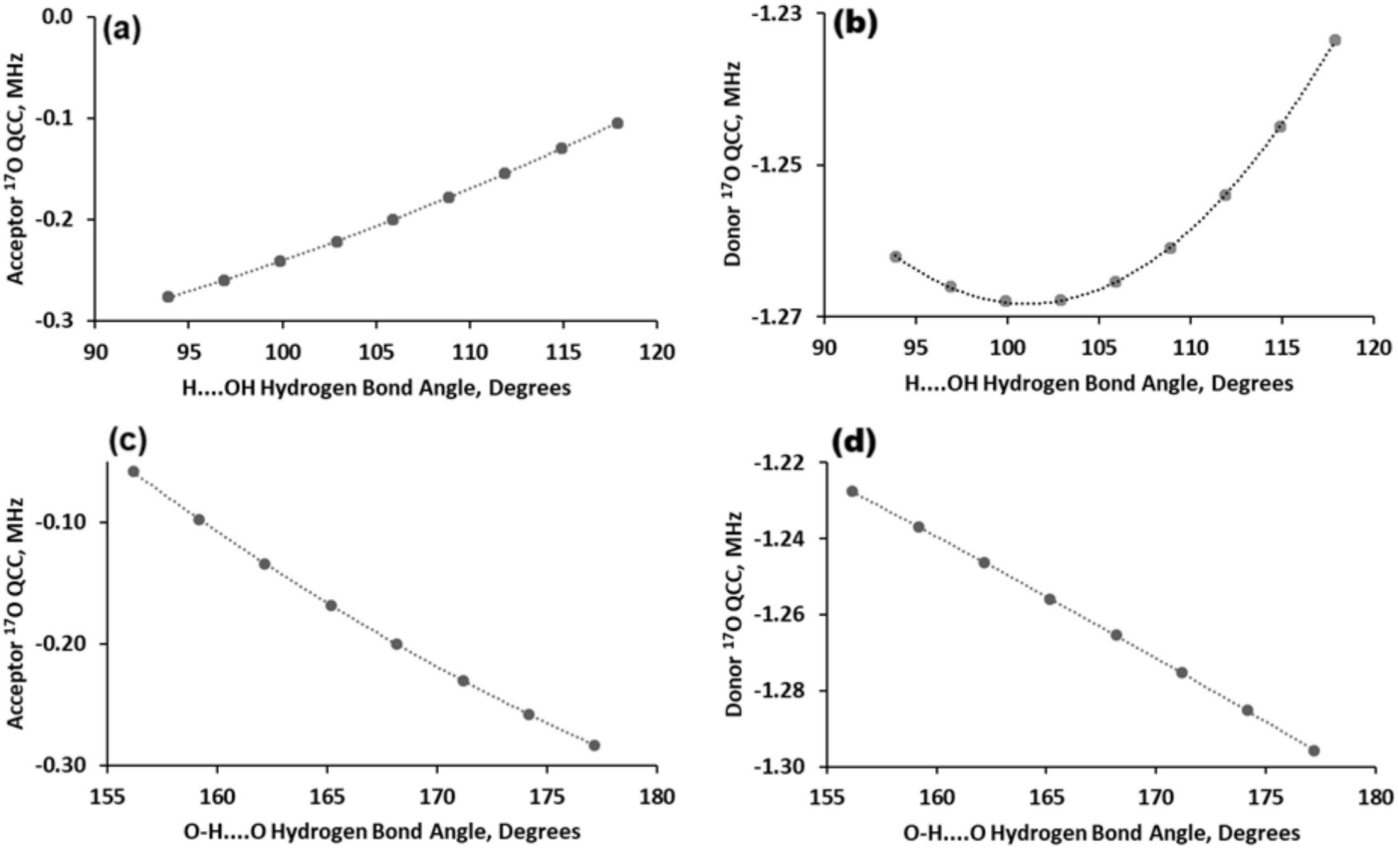
^17^O QCC dependence on hydrogen bond angles in a dimer model. (a) H⋯OH acceptor, (b) H⋯OH donor, (c) OH⋯O acceptor, and (d) OH⋯O donor cases.

### 2.4 QCC Dependence on Internal Geometry

Similarly, variations in the internal geometry of the water molecule, including O–H bond lengths (Figure 4a) and H–O–H bond angles (Figure 4b), produce only minor changes in the QCC. This is in contrast to the ^17^O NMR chemical shift dependence found previously [1]. Although a dependence on these structural parameters is observed, the overall magnitude of the effect remains sufficiently small that it may be considered negligible within the context of hydrogen bonding‐induced variations. For bond length variations, both O–H bonds are modified simultaneously; whereas if only one bond is changing, the effect will be half as much as shown.

**FIGURE 4:**
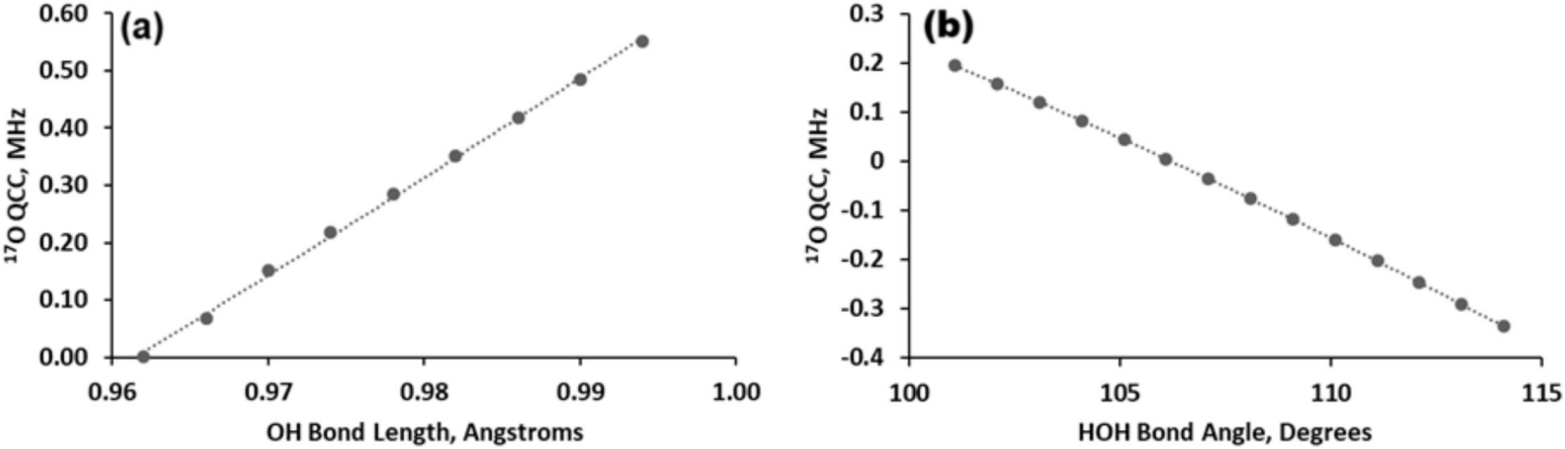
^17^O quadrupole coupling constant (QCC) dependence on the internal geometry of a water molecule. (a) Dependence on O–H bond length, with both O–H bonds varied simultaneously. (b) Dependence on H–O–H bond angle.

As only changes in the hydrogen bond distances produce significant and sufficiently large enough changes in the QCC of the water molecules in the dimer model, only the contribution of this measurable effect in addition to the number and type (accepting or donating) of hydrogen bonding partners is considered when determining the effect of hydrogen bonds to QCC changes in water clusters.

### 2.5 Ice Cluster Models to Determine Additivity of Effects on QCC

Cluster models are used to extract the effects of the number and type of water molecules affecting the ^17^O QCC of a central water molecule. In this work, water clusters derived from the ice structure [28] are employed (see Figure 5). Both first and second nearest‐neighbor effects were considered by extracting (H_2_O)_5_ and (H_2_O)_17_ clusters from the ice structure, respectively.

**FIGURE 5:**
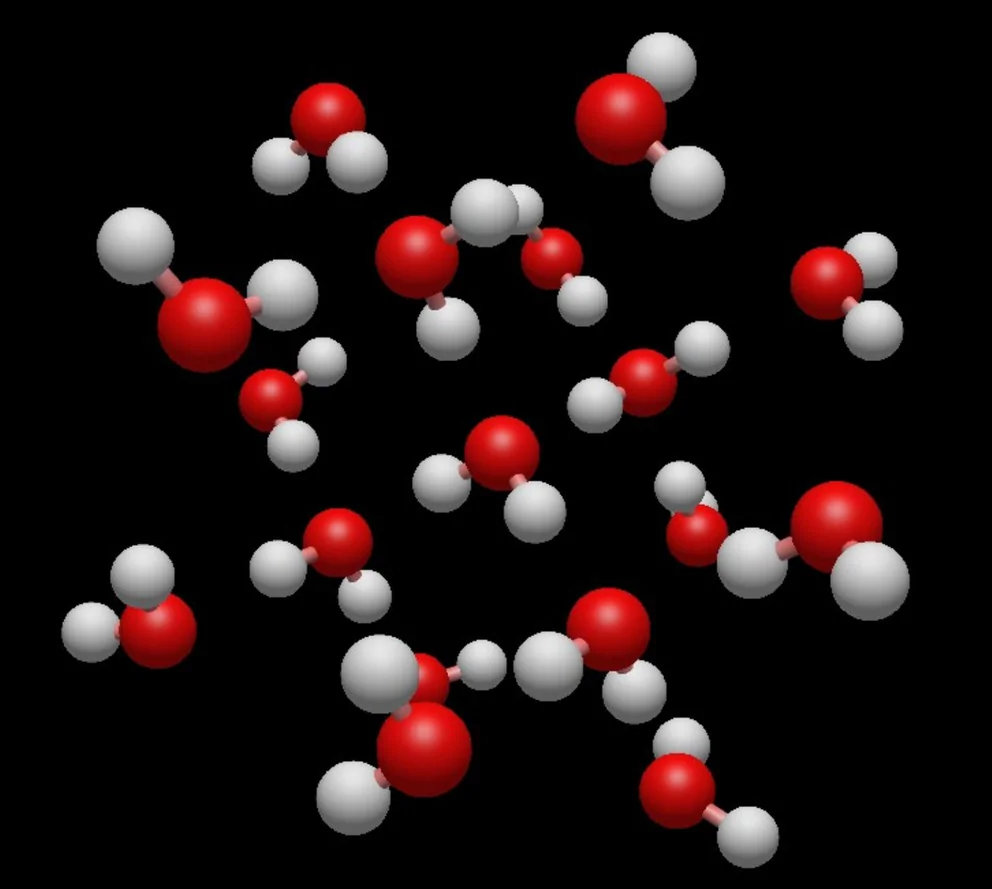
(H_2_O)_17_ extracted from the ice Ih structure [28].

Systematic removal of a water molecule from each cluster is employed to generate new clusters and the QCC of the central molecule is calculated to help determine the first and second neighbor contributions. The neutron structure of ice for (H_2_O)_17_ with the protons' positions geometry optimized at B3LYP/aug‐cc‐pVTZ level gives a QCC of 10.57 MHz for the central molecule without any neighbors. This is then used as a reference for the nearest neighbor effects presented in Table 3. The QCC of the central molecule is calculated for more than 200 fragments derived from (H_2_O)_17_ to cover all possible combinations of first and second nearest neighbors. Predicted values are obtained by using the values presented in Table 3.

For first‐nearest neighbors, a strong correlation is observed between the calculated and predicted ^17^O QCCs for the central water molecule (Figure 6a), demonstrating that first‐shell hydrogen bonding effects are largely additive. Predictive values can be obtained with the assumption that each first‐neighbor hydrogen bond contributes a fixed correction in an additive model depending on its role as either donor or acceptor. Specifically, each donor first neighbor (the central molecule acting as an acceptor) is predicted to contribute −0.30 MHz. On the other hand, each acceptor molecule is assumed to contribute −1.13 MHz. The smaller magnitude in QCC contributions for a donor molecule can be attributed to orbital overlap as explained previously. The close agreement between predicted and explicitly calculated QCC values across different first‐nearest‐neighbor configurations demonstrates that the ^17^O QCC can be effectively expressed as a sum of largely independent hydrogen‐bond contributions. This supports the validity of a first‐nearest‐neighbor additive description of hydrogen bonding effects on the ^17^O EFG in ice.

**FIGURE 6:**
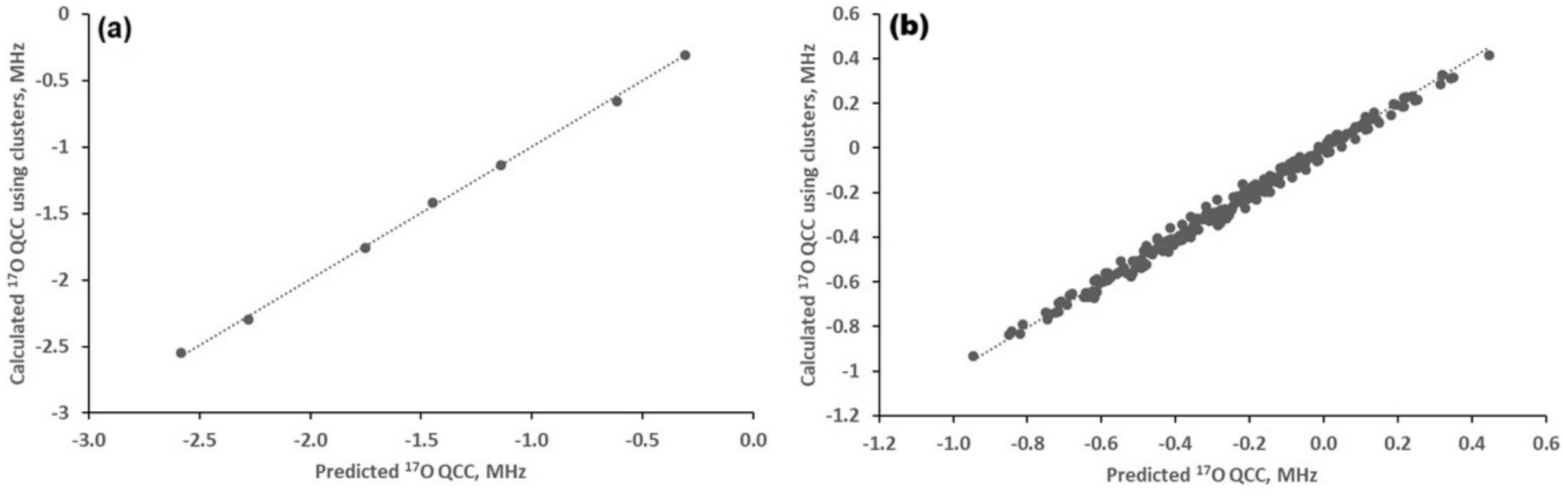
Correlation between calculated and predicted ^17^O QCCs in ice for (a) first nearest neighbor and (b) second nearest neighbor contributions. In both cases, the slope is about 1.0 with a correlation coefficient of 0.99.

Each second nearest neighbor molecule is described in the ice structure depending on whether it acts as a donor or acceptor of a hydrogen bond to the first neighbor that is either a donor or acceptor to the central water molecule. Geometry optimization of the protons at B3LYP/aug‐cc‐pVTZ in the (H_2_O)_17_ ice structure gave a reference QCC of 7.71 MHz for the central molecule without the second neighbors, also described as (H_2_O)_5_. It is worth noting that the large drop in the QCC of the central molecule with the addition of the first nearest neighbors demonstrates their dominant effects. The second nearest neighbors provide additive contributions, as a strong correlation between the calculated and predicted values is once again observed (Figure 6b), with the predicted values being obtained by assuming the second nearest neighbor contributions summarized in Table 3. The differences in the signs for the values of similar magnitude, D and A for example, are dependent on the orientation of the dipole for the second neighbor with respect to the central molecule which creates asymmetry through the shifting electron density to increase or decrease QCC. Additionally, D, A, D* and A* contributions are very similar in magnitude, indicating that at these larger distances only electrostatic effects remain. Because both A* and D are negative and that a donor first neighbor can have two second neighbors as donors (D) but only one acceptor (A), and similarly, an acceptor first neighbor can have two second neighbors as acceptors (A*) but only one donor (D*), the resulting change in QCC is often negative. Averaging over all the ice crystal fragments, the contribution from the second nearest neighbors is −0.26 MHz.

### 2.6 Water Clusters

Ice obviously has an ordered structure. In the liquid phase, hydrogen bond distances as well as orientations are only to be expected to be nonuniform. In order to examine additivity, it is important to test this assumption on water clusters with different hydrogen bond distances and angles. Toward this end, the water clusters [18] afforded by molecular dynamics simulations followed by ab initio geometry optimization are employed. Starting with simply identifying the contributions depending on the type of hydrogen bond (whether a water molecule was acting as an acceptor or donor to the central molecule), and using the values in Table 3, an initially weak or inconsistent correlation (*R* ^2^ = 0.24) between the calculated and predicted QCC values is observed (Figure 7a), indicating that these labels do not completely capture the magnitude across different hydrogen bond environments.

**FIGURE 7:**
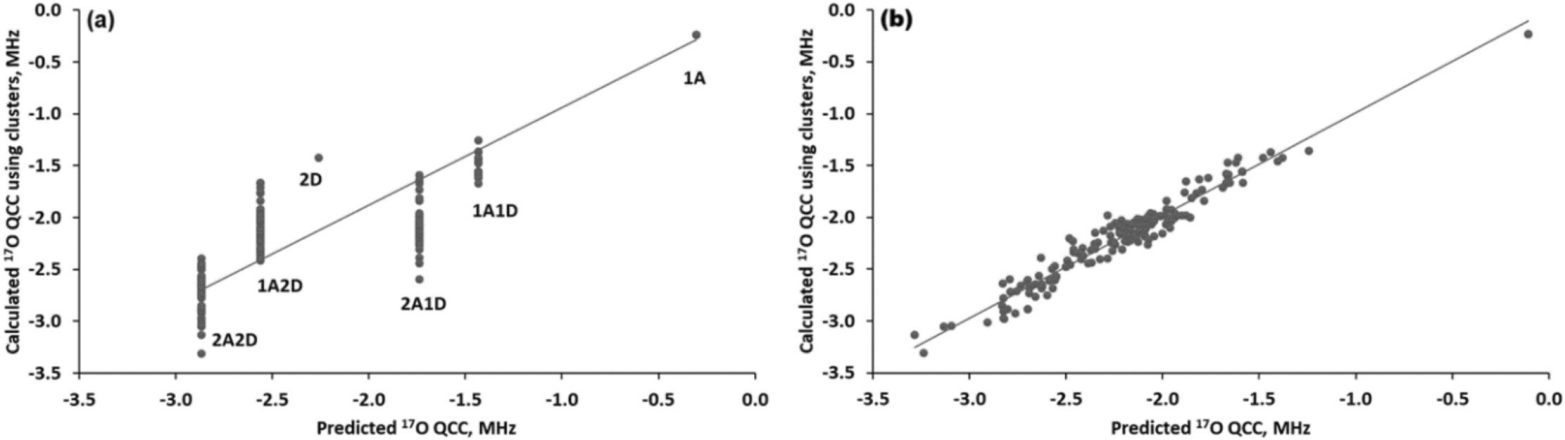
Development of an additive model for ^17^O QCCs in water clusters. Calculated vs. predicted ^17^O QCCs for water clusters containing 3–21 water molecules with geometries obtained from Ref. [18]. In a, first neighbor hydrogen bond partners are only identified as donor or acceptor and predicted changes in QCC are obtained using the values in Table 3 for the nearest neighbor. The labels 1A, 1A1D, 2A1D, 2D, 1A2D, and 2A2D correspond to the number and type (A for acceptor, D for donor) of nearest neighbors. In b, hydrogen bond distance dependence is incorporated using Equations (1) and (2) and contributions from second nearest neighbors are included by using the values in Table 3.

However, adding the changes to QCC that were dependent on hydrogen bond distances produced a significantly stronger correlation (*R* ^2^ = 0.94) with a slope of 1.11 and the addition of second nearest neighbor effects slightly increased the correlation (*R* ^2^ = 0.95), but significantly improved the slope to 0.99 (Figure 7b). An example of these calculations considers the water molecule with its oxygen marked with a star in Figure 8. This water molecule has two nearest neighbors, one accepting a hydrogen bond (labeled 1A) and one donating a hydrogen bond (labeled 1D). Using the hydrogen bond distances, and Equations (1) and (2) yields a contribution of −0.19 MHz from 1D, and −1.23 MHz from 1A. The second nearest neighbors are labeled A* (an acceptor to the first neighbor acceptor 1A) and D (a donor to the first neighbor donor 1D), and using the values in Table 3 for the second nearest neighbors, the additional correction is −0.24 MHz. The total change in the QCC of the water molecule labeled with a star is therefore −1.66 MHz, which satisfactorily agrees with the result using the cluster, 1.60 MHz.

**FIGURE 8:**
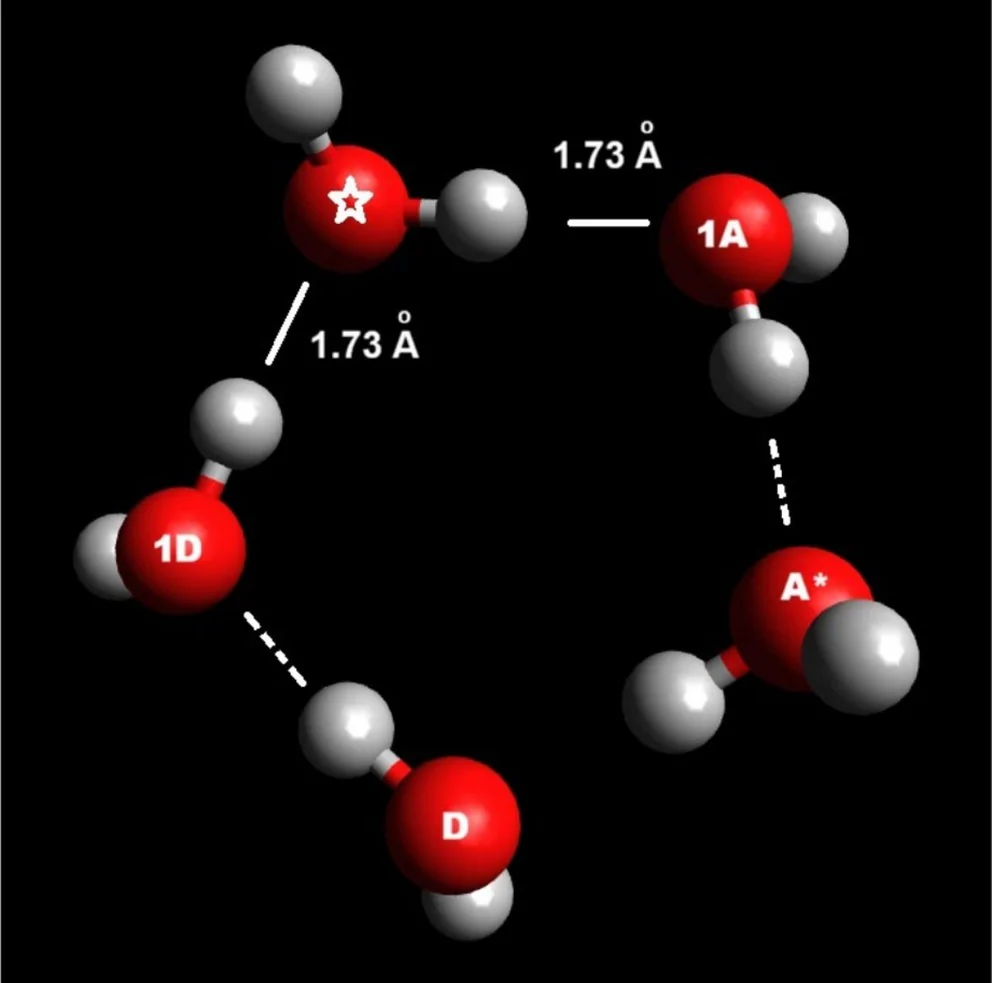
The (H_2_O)_5_ cluster from Ref. [17], showing how first and second nearest neighbors are considered. Prediction of the QCC at the site marked with a star begins with identifying the nearest neighbors (1A and 1D) and their respective hydrogen bond distances. Additional corrections are made for the second nearest neighbors (D and A*).

The summation of the contributions of the second nearest neighbors requires recognition of the fact that first neighbor acceptors can only have one D* and two A* molecules because it is already donating a lone pair to the central molecule, while first neighbor donors can only have one A and two D molecules. Therefore, there are more molecules that contribute a negative shift to the QCC, because both A* and D provide negative contributions, and thus, there is a greater probability that, rather than canceling out, the sum of the second nearest neighbors would be negative, most likely −0.23 MHz. This is likely the main reason why the predicted QCC for the cluster falls short by approximately 0.2 MHz or a slope of 1.10, which is indicative of an overestimation of the QCC by approximately 10% due to the lack of negative contributions. These results demonstrate that ^17^O QCCs in hydrogen‐bonded water networks can be systematically described using an additive framework that includes both first and second nearest neighbor hydrogen bond interactions.

### 2.7 Quadrupole Coupling Tensor

Quadrupolar coupling is a second‐rank tensor. The QCC principal components (*χ*_*ii*_) and asymmetry parameter (*η*) may be expressed in terms of the principal components of the EFG tensor (|*q*_*zz*_| ≥ |*q*_*yy*_| ≥ |*q*_*xx*_|):

(3) $$\chi_{\mathrm{ii}}\;\left(\text{in}\;\mathrm{MHz}\right)=6.01q_{\mathrm{ii}}$$

(4) $$\eta =\left(q_{\mathrm{xx}}–q_{\mathrm{yy}}\right)/q_{\mathrm{zz}},$$

with the EFG tensor components in atomic units. A detailed analysis of the principal components of the quadrupole coupling tensor indicates that the orientation of this second rank tensor remains close to that of an isolated water molecule. The principal component with the largest absolute magnitude, *χ*_*zz*_, is the quadrupolar coupling constant. This remains normal to the plane of the water molecule while the least, *χ*_*xx*_, is on the molecular plane bisecting the HOH bond angle, and the intermediate component, *χ*_*yy*_, also on the molecular plane, is nearly parallel to the line connecting the two hydrogen atoms. As described in this paper, the QCC (*χ*_*zz*_) is sensitive to hydrogen bonding, but the magnitudes of the other components are likewise affected. Similarly, there is greater sensitivity when the water molecule is acting as a hydrogen bond donor. The changes correlate with each other such that the signs and order of the principal components according to their absolute magnitude do not change. The asymmetry parameter, *η*, is affected significantly when the water molecule acts as a donor, but not so much when it is acting as an acceptor. The principal component of largest magnitude, *χ*_*zz*_, decreases slightly faster so the asymmetry parameter increases when hydrogen bonding to an acceptor. In the dimer model, *η* for the donor has increased to 0.88 at a 1.70 Ǻ hydrogen bond distance while for the acceptor, it is 0.74, still very close to the value for an isolated water molecule, 0.76. With (H_2_O)_5_, a similar scenario is observed, as both *χ*_*xx*_ and *χ*_*yy*_ are decreasing in absolute magnitude at the same rate, but not as fast as the decrease in *χ*_*zz*_ when water is acting as a donor. When water is acting as an acceptor, the changes in the three principal components are almost equivalent thus rendering no significant change in the asymmetry parameter. With the clusters extracted from ice, the calculated asymmetry values range from 0.79 to 0.92, with the higher values seen as soon as there is one nearest neighbor acting as an acceptor. Having two nearest neighbors acting as acceptors instead of just one does not change the asymmetry parameter, indicating that for this parameter, additivity does not hold. With the water clusters, the range for the asymmetry parameter is 0.76–0.99, with the high values observed when the hydrogen bond distance to an acceptor is relatively short, 1.6 Ǻ.

It should be noted that the calculated quadrupolar coupling tensors reported in this paper only apply to a static molecule. In the presence of nonisotropic motion, the observed QCC therefore cannot be compared directly to the calculated values presented in this paper. Motional averaging leads to a reduced QCC [9] and the trends described in this current work may only be applicable if the water molecules are exhibiting similar motions.

## 3 Computational Details

Geometry optimizations and EFG calculations were performed using the Gaussian 16 program [29]. Molecular geometries were optimized at the MP2 level of theory [27]. To accurately describe electronic properties such as the EFG, the B3LYP level of theory [25, 26] and the aug‐cc‐pVTZ basis set [21] are used throughout. QCC tensor components (*χ*_*ii*_) were calculated from the EFG tensor components (*q*_*ij*_, in atomic units) via the following conversion, *χ*_*ii*_ = 6.01*q*_*ii*_ MHz [30, 31]. The principal component with the largest absolute magnitude, the QCC, is reported in this paper, *χ*_*zz*_. The level of theory and basis set have been selected as a compromise between computational cost and accuracy, based on basis set convergence tests that showed near‐monotonic behavior at the triple‐zeta level (Table 1). A water cluster consisting of 17 molecules, (H_2_O)_17_ extracted from the neutron structure of ice Ih [28] is used to model the ice environment. Prior to electronic property calculations, the positions of the protons in this cluster are optimized at the MP2 level. A total of 220 unique fragments derived from (H_2_O)_17_ are used for calculating the contributions to QCC from first and second nearest neighbors. For distinct ^17^O sites in hydrogen‐bonded water molecules, clusters from Rakshit et al. [18] are used.

## 4 Conclusions

An additive framework has been developed to describe ^17^O QCCs in hydrogen‐bonded water systems across isolated, clustered, and ice environments. The dimer model establishes that, in addition to the number and type of nearest neighbors, hydrogen‐bond distance is the primary geometric factor governing QCC variations, while angular and intramolecular structural effects are comparatively minor and can be ignored.

Building on this, ice cluster models are used to isolate the effects of hydrogen‐bond connectivity under equidistant conditions. First‐nearest‐neighbor interactions are found to dominate the QCC shifts of the central water molecule. Because the change in the QCC is smaller when a water molecule acts as an acceptor than when it acts as a donor, expected changes in QCC are not simple multiples of the number of hydrogen bond partners. Nonetheless, first‐nearest‐neighbor contributions are still well described by an additive scheme based on donor and acceptor contributions. Furthermore, based on water cluster calculations, classification based solely on donor or acceptor roles is insufficient to reproduce QCC trends. The inclusion of both hydrogen‐bond distance dependence, as provided by Equations (1) and (2), and second‐nearest‐neighbor effects yield a much more robust and transferable description of ^17^O QCC variations. Second‐nearest‐neighbor interactions introduce smaller but systematic corrections. These contributions arise from asymmetry in hydrogen‐bond connectivity and produce a consistent net decrease in the QCC. Inclusion of these effects reduces the deviation between model predictions and full electronic structure calculations on water clusters and restores near‐quantitative agreement across all cluster sizes considered.

Overall, the results demonstrate that QCCs in water are governed by a hierarchy of hydrogen‐bond interactions that are effectively additive when appropriately parameterized. Finally, because only the identification of hydrogen bonds and computation of hydrogen bond distances are required, the proposed model described in this paper provides a computationally efficient and physically interpretable framework for estimating electric field gradients in extended aqueous environments, enabling direct comparison between molecular simulations and the ^17^O quadrupole coupling constant.

## Author Contributions

**Nabeeha Haque:** writing – original draft, software, investigation. **Alexander G. de Dios:** software, validation, visualization, investigation. **Angel C. de Dios:** conceptualization, writing – review and editing, project administration, supervision, resources, software, formal analysis, methodology, investigation, validation, visualization.

## Conflicts of Interest

The authors declare no conflicts of interest.

## Data Availability

Data sharing is not applicable to this article as no datasets were generated or analyzed during the current study.

## References

[1] A. C. de Dios , “ ^17^O Chemical Shifts in Water,” Journal of Physical Chemistry A 129, no. 48 (2025): 11203–11212, 10.1021/acs.jpca.5c07170. PMC12683631 41277398

[2] Q. Zhang , E. Y. Chekmenev , and R. J. Wittebort , “ ^17^O Quadrupole Coupling and Chemical Shielding Tensors in an H‐Bonded Carboxyl Group: α‐Oxalic Acid,” Journal of the American Chemical Society 125, no. 30 (2003): 9140–9146, 10.1021/ja034495e. 15369371

[3] G. Wu , “Solid‐State ^17^O NMR Studies of Organic and Biological Molecules,” Progress in Nuclear Magnetic Resonance Spectroscopy 52, no. 2–3 (2008): 118–169, 10.1016/j.pnmrs.2007.07.004. 31779879

[4] S. D. McCalpin , R. Fu , T. Ravula , G. Wu , and A. Ramamoorthy , “Magnetically Aligned Nanodiscs Enable Direct Measurement of ^17^O Residual Quadrupolar Coupling for Small Molecules,” Journal of Magnetic Resonance 346 (2023): 107341, 10.1016/j.jmr.2022.107341. PMC11585078 36473327

[5] B. B. Garrett , A. B. Denison , and S. W. Rabideau , “Oxygen‐17 Relaxation in Water,” Journal of Physical Chemistry 71, no. 8 (1967): 2606–2611, 10.1021/j100867a033.

[6] Y. Levy and J. N. Onuchic , “Water and Proteins: A Love‐Hate Relationship,” Proceedings of the National Academy of Sciences of the United States of America 101, no. 10 (2004): 3325–3326, 10.1073/pnas.0400157101. PMC373459 14993602

[7] D. C. Robinson Brown , T. R. Webber , T. M. Casey , J. Franck , M. S. Shell , and S. Han , “Computation of Overhauser Dynamic Nuclear Polarization Processes Reveals Fundamental Correlation Between Water Dynamics, Structure, and Solvent Restructuring Entropy,” Physical Chemistry Chemical Physics 26, no. 20 (2024): 14637–14650, 10.1039/d4cp00030g. PMC12505230 38742831

[8] C. Takehiko , “Deuteron Magnetic Resonance Study of Some Crystals Containing an O—D···O Bond,” Journal of Chemical Physics 41, no. 5 (1964): 1352–1358, 10.1063/1.1726072.

[9] E. G. Keeler , V. K. Michaelis , and R. J. Griffin , “17O NMR Investigation of Water Structure and Dynamics,” Journal of Physical Chemistry B 120, no. 32 (2016): 7851–7858, 10.1021/acs.jpcb.6b05755. PMC5418864 27454747

[10] V. K. Michaelis , E. G. Keeler , T.‐C. Ong , et al., “Structural Insights Into Bound Water in Crystalline Amino Acids: Experimental and Theoretical 17O NMR,” Journal of Physical Chemistry B 119, no. 25 (2015): 8024–8036, 10.1021/acs.jpcb.5b04647. PMC4894719 25996165

[11] S. Dong , K. Yamada , and G. Wu , “Oxygen‐17 Nuclear Magnetic Resonance of Organic Solids,” Zeitschrift für Naturforschung A 55, no. 1–2 (2000): 21–28, 10.1515/zna-2000-1-205.

[12] F. Xie , D. S. Tikhonov , and M. Schnell , “Electric Nuclear Quadrupole Coupling Reveals Dissociation of HCl With a Few Water Molecules,” Science 384, no. 6703 (2024): 1435–1440, 10.1126/science.ado7049. 38843353

[13] J. Verhoeven , H. Bluyssen , and A. Dymanus , “Hyperfine Structure of HDO and D2O by Beam Maser Spectroscopy,” Physics Letters A 26, no. 9 (1968): 424–425, 10.1016/0375-9601(68)90256-9.

[14] T. S. Pennanen , J. Vaara , P. Lantto , A. J. Sillanpää , K. Laasonen , and J. Jokisaari , “Nuclear Magnetic Shielding and Quadrupole Coupling Tensors in Liquid Water: A Combined Molecular Dynamics Simulation and Quantum Chemical Study,” Journal of the American Chemical Society 126, no. 35 (2004): 11093–11102, 10.1021/ja048049i. 15339196

[15] D. T. Edmonds and A. Zussman , “Pure Quadrupole Resonance of 17O in Ice,” Physics Letters A 41, no. 2 (2002): 167–169, 10.1016/0375-9601(72)91097-3.

[16] T. S. Pennanen , P. Lantto , A. J. Sillanpää , and J. Vaara , “Nuclear Magnetic Resonance Chemical Shifts and Quadrupole Couplings for Different Hydrogen‐Bonding Cases Occurring in Liquid Water: A Computational Study,” Journal of Physical Chemistry A 111, no. 1 (2006): 182–192, 10.1021/jp065507w. 17201402

[17] R. Car and M. Parrinello , “Unified Approach for Molecular Dynamics and Density‐Functional Theory,” Physical Review Letters 55, no. 22 (1985): 2471–2474, 10.1103/physrevlett.55.2471. 10032153

[18] A. Rakshit , P. Bandyopadhyay , J. P. Heindel , and S. S. Xantheas , “Atlas of Putative Minima and Low‐Lying Energy Networks of Water Clusters N = 3–25,” Journal of Chemical Physics 151, no. 21 (2019): 214307, 10.1063/1.5128378. 31822087

[19] M. Alfredsson and K. Hermansson , “Hartree–Fock and DFT Calculations of Quadrupole Coupling Constants in Water Clusters and Ice,” Chemical Physics 242, no. 2 (1999): 161–175, 10.1016/s0301-0104(99)00012-9.

[20] T. H. Dunning, Jr. , “Gaussian Basis Sets for Use in Correlated Molecular Calculations. I. The Atoms Boron Through Neon and Hydrogen,” Journal of Chemical Physics 90 (1989): 1007–1023, 10.1063/1.456153.

[21] R. A. Kendall , T. H. Dunning, Jr. , and R. J. Harrison , “Electron Affinities of the First‐Row Atoms Revisited. Systematic Basis Sets and Wave Functions,” Journal of Chemical Physics 96 (1992): 6796–6806, 10.1063/1.462569.

[22] D. E. Woon and T. H. Dunning, Jr. , “Gaussian Basis Sets for Use in Correlated Molecular Calculations. III. The Atoms Aluminum Through Argon,” Journal of Chemical Physics 98 (1993): 1358–1371, 10.1063/1.464303.

[23] K. A. Peterson , D. E. Woon , and T. H. Dunning, Jr. , “Benchmark Calculations With Correlated Molecular Wave Functions. IV. The Classical Barrier Height of the H+H_2_ → H_2_+H Reaction,” Journal of Chemical Physics 100 (1994): 7410–7415, 10.1063/1.466884.

[24] J. F. Hinton , P. Guthrie , P. Pulay , and K. Wolinski , “Ab Initio Quantum Mechanical Calculation of the Chemical Shift Anisotropy of the Hydrogen Atom in the (H_2_O)_17_ Water Cluster,” Journal of the American Chemical Society 114, no. 5 (1992): 1604–1605, 10.1021/ja00031a010.

[25] A. D. Becke , “Density‐Functional Thermochemistry. III. The Role of Exact Exchange,” Journal of Chemical Physics 98, no. 7 (1993): 5648–5652, 10.1063/1.464913.

[26] C. Lee , W. Yang , and R. G. Parr , “Development of the Colle‐Salvetti Correlation‐Energy Formula Into a Functional of the Electron Density,” Physical Review B 37 (1988): 785–789, 10.1103/PhysRevB.37.785. 9944570

[27] M. J. Frisch , M. Head‐Gordon , and J. A. Pople , “A Direct MP2 Gradient Method,” Chemical Physics Letters 166, no. 3 (1990): 275–280, 10.1016/0009-2614(90)80029-D.

[28] V. M. Nield and R. W. Whitworth , “The Structure of Ice Ih From Analysis of Single‐Crystal Neutron Diffuse Scattering,” Journal of Physics: Condensed Matter 7 (1995): 8259–8271, 10.1088/0953-8984/7/43/006.

[29] Gaussian 16, Revision A.03 2016. ‘M. J. Frisch, G. W. Trucks, H. B. Schlegel, G. E. Scuseria, M. A. Robb, J. R. Cheeseman, G. Scalmani, V. Barone, G. A. Petersson, H. Nakatsuji, X. Li, M. Caricato, A. V. Marenich, J. Bloino, B. G. Janesko, R. Gomperts, B. Mennucci, H. P. Hratchian, J. V. Ortiz, A. F. Izmaylov, J. L. Sonnenberg, D. Williams‐Young, F. Ding, F. Lipparini, F. Egidi, J. Goings, B. Peng, A. Petrone, T. Henderson, D. Ranasinghe, V. G. Zakrzewski, J. Gao, N. Rega, G. Zheng, W. Liang, M. Hada, M. Ehara, K. Toyota, R. Fukuda, J. Hasegawa, M. Ishida, T. Nakajima, Y. Honda, O. Kitao, H. Nakai, T. Vreven, K. Throssell, J. A. Montgomery, Jr., J. E. Peralta, F. Ogliaro, M. J. Bearpark, J. J. Heyd, E. N. Brothers, K. N. Kudin, V. N. Staroverov, T. A. Keith, R. Kobayashi, J. Normand, K. Raghavachari, A. P. Rendell, J. C. Burant, S. S. Iyengar, J. Tomasi, M. Cossi, J. M. Millam, M. Klene, C. Adamo, R. Cammi, J. W. Ochterski, R. L. Martin, K. Morokuma, O. Farkas, J. B. Foresman, and D. J. Fox.’ Gaussian, Inc., Wallingford CT.

[30] W. Scheubel , H. Zimmermann , and U. Haeberlen , “17O Quadrupole Coupling and Nuclear Magnetic Shielding Tensors in Benzophenone,” Journal of Magnetic Resonance 3, no. 3 (1985): 544–555, 10.1016/0022-2364(85)90244-6.

[31] P. Pyykkö , “The Nuclear Quadrupole Moments of the 20 First Elements: High‐Precision Calculations on Atoms and Small Molecules,” Zeitschrift für Naturforschung A 47, no. 1–2 (1992): 189–196, 10.1515/zna-1992-1-233.

